# Colon Adenocarcinoma Metastasis Through Ileocolic Fistula to Small Bowel in the Setting of Crohn's Disease

**DOI:** 10.14309/crj.0000000000000925

**Published:** 2022-11-24

**Authors:** Michael T. Tseng, Taseen Syed, Patricija Zot, Ravi Vachhani

**Affiliations:** 1 Department of Internal Medicine, Virginia Commonwealth University, Richmond, VA; 2 Department of Gastroenterology, Nutrition and Transplant Hepatology, Virginia Commonwealth University, Richmond, VA; 3 Department of Gastroenterology, Hunter Holmes McGuire Veterans Affairs Medical Center, Richmond, VA; 4 Department of Pathology, Virginia Commonwealth University, Richmond, VA

## Abstract

Patients with Crohn's disease are at higher risk of developing colorectal cancer and gastrointestinal fistula. Few cases in the past described colorectal cancer metastasized within the gastrointestinal tract through a fistula. We report a case of sigmoid colon adenocarcinoma in a patient with Crohn's disease that metastasized to the ileum through an ileocolic fistula tract. In addition to presenting a unique pathological phenomenon in these patients, this case raises awareness of the importance of regular follow-up and early initiation of inflammatory bowel disease therapies.

## INTRODUCTION

Colorectal cancer (CRC) is the third most common type of cancer worldwide, with colon cancer more frequent than rectal cancer.^1^ Patients with inflammatory bowel disease (IBD) are 2–6 times more likely to develop CRC than the general population.^2^ Risk of CRC in Crohn's disease (CD) is increased with the extent and duration of the disease; therefore, guidelines recommend to start surveillance colonoscopy in patients who have 30% or more of colon involvement after 8 years of disease.^3^ The most common site of colon cancer metastasis is the liver because of its anatomical location and the presence of portal circulation.^4^ When metastasized, the most common route is hematogenous or localized spread.^5^ We report an unprecedented case of adenocarcinoma of the sigmoid colon in a patient with CD that metastasized to the small bowel through an ileocolic fistula tract.

## CASE REPORT

**Figure 1.**
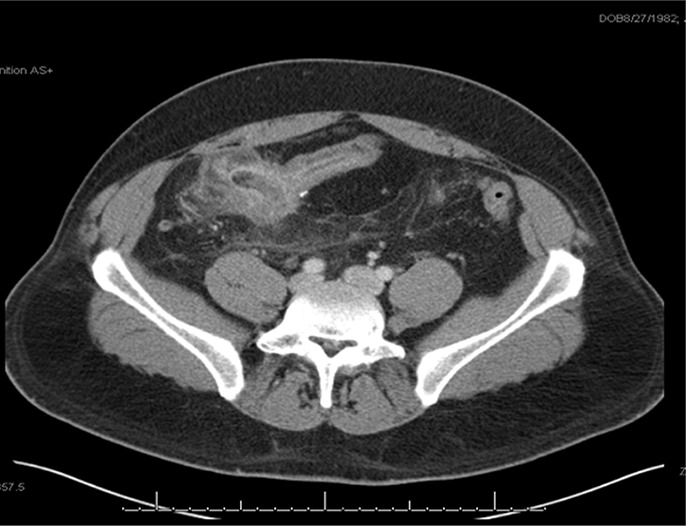
Abdominal computed tomography: thickened terminal ileum immediately adjacent to the sigmoid colon (shown by arrow) with no clear fat plane between bowel loops, which represents a fistula formation. Measurement 31.5 × 41.5 mm.

**Figure 2.**
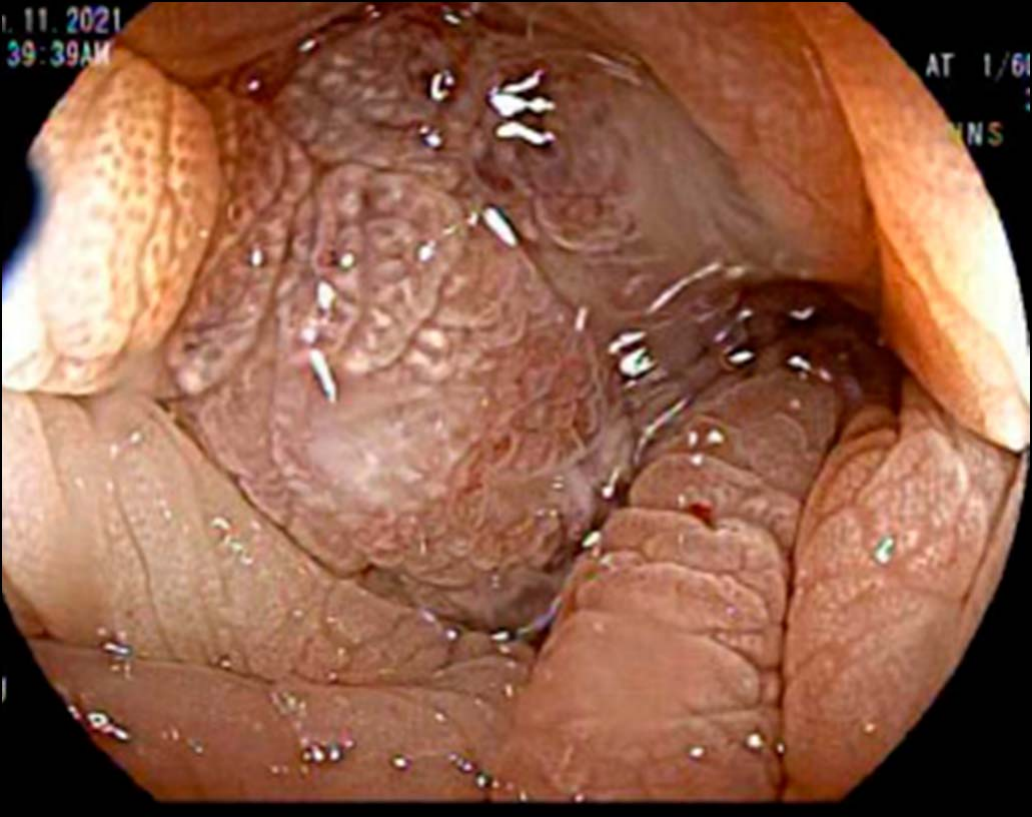
Colonoscopy: stricture at the distal sigmoid colon unable to traverse with the colonoscope.

**Figure 3.**
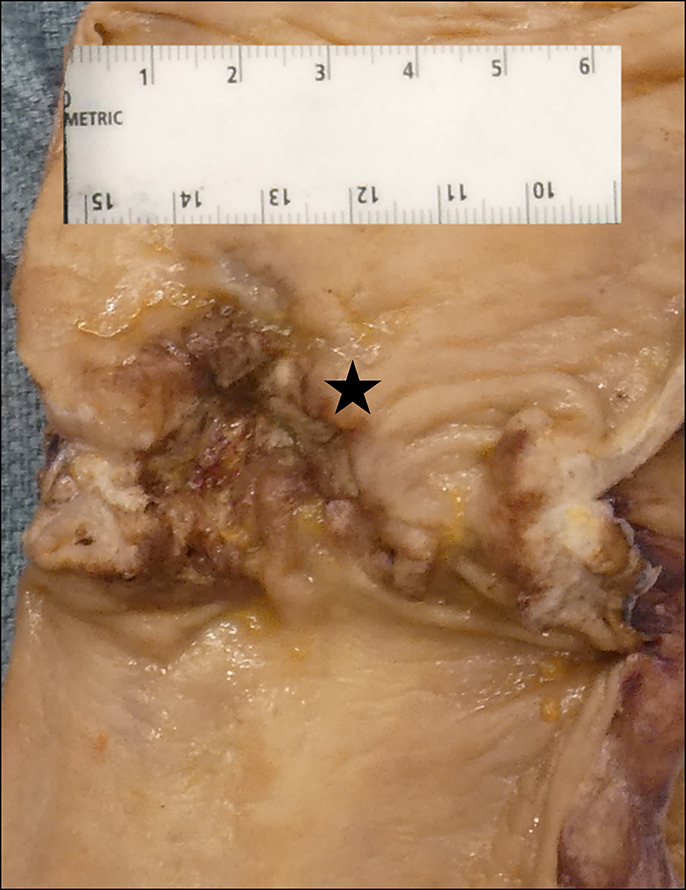
Gross specimen included a portion of the terminal ileum, attached appendix, colon, rectum, and anus. On the serosal surface, a fistula was identified at the ileocecal valve. The specimen was opened to reveal a 3.0 × 3.0 cm ulcerated concentric mass (asterisk) with a central area of tan-gray necrosis.

**Figure 4.**
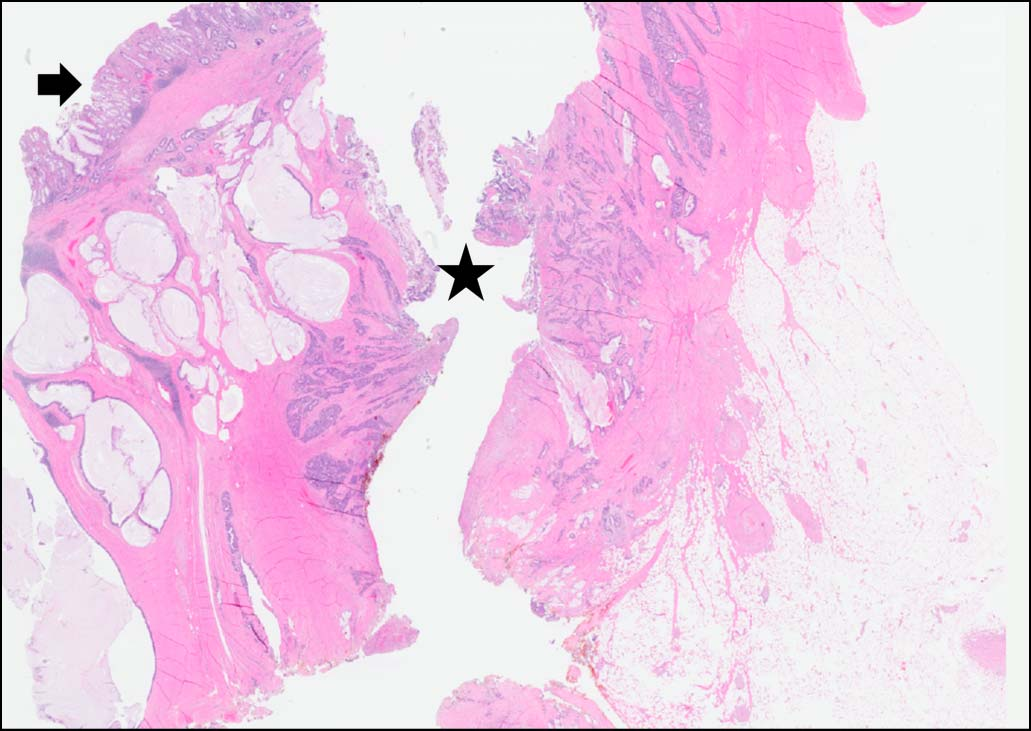
Moderately differentiated adenocarcinoma with mucinous features (the same morphology as in the sigmoid colon specimen) is found in the adjacent terminal ileum (normal ileum mucosa shown by arrow) at the site of fistula (asterisk).

A 37-year-old White man with a history of ileocolic CD presented with abdominal pain associated with nausea, vomiting, and diarrhea. The patient was diagnosed with CD at age of 19 years old and previously on mesalamine, but was nonadherent to treatment because of intolerance, thus required intermittent prednisone tapers during flares. Most recent colonoscopy before admission was conducted in October 2011 with pseudopolyps and small erosion noted in the sigmoid colon and cecum and without extensive colonic involvement of CD documented. On admission, the patient was hemodynamically stable and afebrile, with a blood pressure of 130/80 mm Hg, a heart rate of 90 beats per minute, and a respiratory rate of 14 per minute. Physical examination was remarkable for diffuse abdominal tenderness and hypoactive bowel sounds. The patient had abdominal computed tomography that revealed prominent wall thickening of the terminal ileum consistent with active inflammation suggestive of a CD flare with a fistulous tract noted between the terminal ileum and the sigmoid colon (Figure 1). Surgery was deferred by colorectal surgery services because of limited evidence of rectal bleeding, abscess, perforation, obstruction or peritoneal signs; thus, the patient was started on budesonide with a plan to start infliximab as an outpatient for fistulizing CD. Colonoscopy before initiating infliximab showed a severe rectosigmoid stricture that could not be traversed with a colonoscopy (Figure 2). Biopsies from the stricture revealed adenocarcinoma of the colon. The patient underwent open total proctocolectomy; however, at the time of surgery, there was involvement of the terminal ileum through the sigmoid colon-ileum fistulous tract, and therefore, a proximal portion of the terminal ileum was also surgically resected (Figure 3). The pathology showed moderately differentiated adenocarcinoma with a mucinous feature of the sigmoid colon with the involvement of the terminal ileum at the fistula site (Figure 4). The patient recovered well from surgery and is currently undergoing adjuvant chemotherapy.

## DISCUSSION

We have reported an unusual case of colon cancer in a young patient with CD that metastasized through a fistulous tract to the terminal ileum. Prior reported cases of intraintestinal tract metastasis through fistula are almost always through an anal fistula or in closer proximity to the anatomical position.^6–9^ Chronic intestinal inflammation such as IBD leads to a higher incidence of gastrointestinal malignancy.^10^ The risk of small bowel adenocarcinoma is higher than CRC in patients with IBD, with an incidence ratio of 5.7 and 27.1 for CRC and small bowel adenocarcinoma, respectively.^11,12^ Despite his young age, our patient developed colon cancer, which was likely secondary to ongoing accelerated inflammation in the setting of noncompliance with medical therapy.

Patients with CD are more prone to fistula formation with occurrence reported in 17%–50% of patients with CD. The cumulative incidence of fistulizing CD is 21% 1 year after diagnosis that is increased to 50% after 20 years.^13^ An ileal-sigmoid fistula was first described in a surgical case report by Hurwitt and Lentino in 1957,^14^ and subsequent cases of ileocolic fistulas were noted from surgical complications or radiotherapy.^15^ Common metastatic sites of colon cancers include the liver, lungs, nervous system, and peritoneum.^5^ Intragastrointestinal tract metastasis is also described in a few cases in the past such as a sigmoid adenocarcinoma discovered in the duodenal bulb by Iwamuro et al.^16^ The mechanism of intragastroinestinal tract metastasis is suspected to be through hematogenous, translocation of tumor cells, or peritoneal spread.^5^ Treatment of the ileocolic fistula is often surgical because they are less likely to resolve spontaneously. However, treatment is individualized for each patient because the morphology of the fistula and course are often complicated. In our patient, in addition to surgical treatment, his extensive disease warranted chemotherapy.

In conclusion, CD is associated with a higher risk of fistula development. Few cases in the past described CRC metastasizing within the gastrointestinal tract through a fistula. Intriguingly in our case, sigmoid adenocarcinoma developed and further metastasized to the ileum through the ileal-sigmoid fistula in the setting of CD. In addition to presenting a unique pathological phenomenon in patients with IBD, this case raises awareness of the importance of regular follow-up and early initiation of IBD therapies.

## DISCLOSURES

Author contributions: M. Tseng, T. Syed, and R. Vachhani conceptualized and prepared the manuscript. P. Zot prepared the pathology report. All authors revised and approved the final version. M. Tseng is the article guarantor.

Financial disclosure: None to report.

Previous presentation: American College of Gastroenterology 2021 Virtual Meeting; October 2021; Las Vegas, Nevada.

Informed consent was obtained for this case report.
